# Effectiveness of Video-Based Online Training for Health Care Workers to Prevent COVID-19 Infection: An Experience at a Tertiary Care Level Institute, Uttarakhand, India

**DOI:** 10.7759/cureus.14785

**Published:** 2021-05-01

**Authors:** Rakesh Sharma, Aroop Mohanty, Vanya Singh, Vishwas A S, Puneet K Gupta, Prasuna Jelly, Pratima Gupta, Shalinee Rao

**Affiliations:** 1 College of Nursing, All India Institute of Medical Sciences, Rishikesh, IND; 2 Microbiology, All India Institute of Medical Sciences, Gorakhpur, IND; 3 Microbiology, All India Institute of Medical Sciences, Rishikesh, IND; 4 Microbiology, All India Institute of Medical Sciences, Bilaspur, IND; 5 Microbiology, All India Institute of Medical Sciences, .Rishikesh, IND; 6 Pathology, All India Institute of Medical Sciences, Rishikesh, IND

**Keywords:** covid-19, infection control practices, health care workers, training, virtual

## Abstract

Purpose

Amidst the current COVID-19 pandemic, traditional teaching methodology took a back foot. However, an urgent need for training health care worker (HCW) on preventive measures for COVID-19 infection was the need of the hour. Keeping in mind the precautionary measures required to combat COVID-19 infection, the only promising option for training was by adopting an online learning method. This study was undertaken to determine the effectiveness of video-based training using online platforms for infection prevention and control (IPC) training during the COVID-19 pandemic.

Methods

A quasi-experimental study, with only one experimental group comprising of HCWs, was undertaken to assess the effect of intervention which included video-assisted teaching-learning regarding IPC measures with a special focus on COVID-19 at a tertiary care Institute in North India. Online sessions were conducted on Do’s and Don’ts, Hand Hygiene, donning and doffing of personal protective equipment (PPE), cleaning and disinfection, and bio-medical waste (BMW) management with the help of pre-recorded videos which was pre-validated. The session was followed by online interaction with participants by a single resource person. Pre-test and post-test were conducted through google forms before commencement and at the end of the online session respectively. The data were analyzed in terms of descriptive frequencies and percentages of different domains to describe the pre- and post-test knowledge.

Results

A total of 576 participants were included in the study. There was a statistically significant gain in scores of all questions related to cleaning and disinfection; and BMW. No significant change was observed in knowledge regarding the sequence of doffing (p-value: 0.94). The result of pairwise comparisons pre-test and post-test scores showed that intervention through video-assisted teaching-learning resulted in improvement of knowledge which was found to be statistically significant (p-value < 0.001).

Conclusion

Video-assisted teaching-learning through virtual platforms effectively trained health personnel on infection prevention and control practices during the COVID-19 pandemic. Video-assisted training can successfully be handled by a single resource person to impart the knowledge and skill to the trainee. Virtual teaching and learning is a feasible and efficient method to deliver training to HCWs on infection control practices and this methodology may be adopted in the future for several other training in manpower crunch situations, similar restrictive circumstances as posed by the COVID-19 pandemic.

## Introduction

The challenges created by Coronavirus disease (COVID-19) pandemic has affected the wellbeing of all individuals directly or indirectly globally [1,2]. This highly infectious disease has already overshadowed the other pandemics in the recent past caused by similar coronaviruses like SARS and Middle East Respiratory Syndrome (MERS) CoV. It has placed unparalleled stress on all the health care workers (HCWs) worldwide and has therefore highlighted the necessity for strict infection control measures in health care facilities to prevent the nosocomial transmission of this deadly virus [3]. An integral component of this workforce responding to this global pandemic, are the HCWs and they not only should have comprehensive theoretical knowledge and practical skills but must also implement them in regular practice for infection prevention and control [4]. Appropriate training will facilitate the acquisition of required knowledge and skill to make them competent and efficient in performing their assigned roles. During disease outbreaks, a sudden need for teaching and training large numbers of HCWs for capacity building came under the radar [5,6].

Past experiences during the severe acute respiratory syndrome (SARS) outbreak, the 2009 H1N1 influenza, and the present situation posed by COVID-19 pandemics have highlighted the need for infection control practices especially appropriate use of personal protective equipment (PPE) for the protection of HCWs against these dangerous respiratory infections [7-10]. Safe donning and doffing of PPE is an essential part of protection for the HCWs, and its appropriate use may help to downscale the potential of communicable disease to be transmitted from and to patients, families, and therefore the public [11,12].

The COVID-19 pandemic has affected educational systems in countries around the world and led to the closure of face-to-face courses in schools as well as universities, including medical schools [13]. Social distancing in pandemic times has prevented physical live educational activities. Educational sessions regarding the appropriate use of PPE were not feasible due to the restrictions of large in-person skills sessions. Yet, an urgent need for PPE training was expressed by the HCWs of different disciplines, especially due to the availability of conflicting recommendations available for PPE removal as well as the availability of different types of masks and PPE, leading to confusion among HCWs [14].

Considering the urgent need to train the HCWs, and the limitation of space and maintaining social distance, virtual education was seen as the only available option for preparing all the HCWs regarding safe infection prevention and control (IPC) practices with a focus on COVID-19 and was observed as a turning point for educating the HCWs. [15]. Virtual learning offers many benefits over physical live classes, such as accessibility and availability around the clock, asynchronous discussions with classmates, immediate feedback on tests, and flexibility. Moreover, once established, they are cost-effective and become a time and resource-saving approach compared to traditional teaching techniques. However, despite the benefits of virtual learning, it is not an easy task to be implemented. To the best of our knowledge, there have been no documented studies analyzing the use of video-based learning via a virtual platform for training of HCWs regarding IPC practices with special instructions regarding prevention from COVID-19. The aim of this study was to determine the effectiveness of an online training program using video-assisted teaching-learning through a virtual platform in improving the knowledge and skills of HCWs regarding IPC during the COVID-19 pandemic. In addition, to also identify the online course components that were most effective in promoting such learning. Further results of this study will help us in knowing and understanding the training needs of the HCWs regarding the implementation of IPC in the hospital, especially during this current COVID-19 pandemic and for future practices and provision of additional learning if deficits arise.

## Materials and methods

This was a quasi-experimental study, with only one experimental group comprising of HCWs. The intervention given to this group was video-assisted teaching-learning regarding IPC measures with a special focus on COVID-19. This study was conducted at a 900 bedded tertiary care institute in the Northern part of India from April to May 2020. At the time of the study, about 200 beds were clearly demarcated for admitting COVID-19 patients at this Institute. This was facilitated by the hospital infection control team (HICT) in collaboration with the faculty of both medical and nursing college and coordinated by a central unit of Advance Center of Continous Professional Development. Using a convenience sampling method, 575 study participants, consisting of nursing officers, technicians, and residents, were included. A semi-structured questionnaire was used to collect all the data. The participants who did not either give the pre-or post-test or did not attend the entire session were excluded from the study. Informed consent was obtained from each participant before undertaking the study. The Institute Ethics Committee also approved the study (letter no. 209/IEC/IM/NF/2020).

Video-assisted online sessions were conducted on Do’s and Don’ts, Hand Hygiene, donning and doffing of PPE, cleaning and disinfection, and bio-medical waste (BMW) management with the help of pre-recorded videos which was pre-validated. The virtual live training sessions were conducted by members of the IPC team. The content of the Powerpoint presentation and pre-recorded videos used were prepared as per the Institute’s IPC policy and procedures formulated based on guidelines of the World Health Organization (WHO), Centre for Disease Control (CDC), Ministry of Health and Family Welfare, and Indian Council of Medical Research (ICMR). Each training was of 1 hour and was preceded by a pre-test questionnaire distributed to the participants 15 min before the session. The subject expert briefed each session, and after a formal introduction, specific topic-related PPT and videos were shared on live platforms using the ZOOM Meeting application (Table 4, Appendix). Each training session was conducted for 20-25 HCWs and followed by online interaction and discussion done by a single trainer with all participating trainees who clarified all doubts raised by the participants. Pre-test and post-test were conducted through google forms before commencement and at the end of the online session, respectively, using the same structured questionnaire.

A Google-based questionnaire, used as a tool for assessment encompassed five sections were as follows:

· Segment-1: This section of the questionnaire included the Do’s and Don’ts regarding basic cough etiquette and measures required to prevent virus spread.

· Segment-2: This section concerned the most fundamental aspect of IPC, i.e., Hand Hygiene and consisted of all the basic aspects of hand rub, hand wash, and duration of each.

· Segment-3: This section was regarding the PPE in which the main emphasis was on the correct sequence of donning and doffing of the same.

· Segment-4: This consisted of the fundamental questions asked in cleaning and disinfection.

· Segment-5: The last section was about the latest rules and regulations of BMW management.

Participants were requested to fill the feedback form comprising of a close-ended questionnaire using a 3-point Likert scale and responses were recorded to evaluate perceptions of participants on video-based training delivered through a virtual live platform.

Data were entered using the data validation feature of Microsoft professional plus Excel 2016 to maintain the data quality from Google sheets and exported to IBM SPSS Statistics for Windows, version 23 (IBM Corp., Armonk, NY, USA) for the analysis. Shapiro-Wilk test was used to check the normality of data. The data were analyzed in terms of descriptive frequencies and percentages of different segments to describe the pre-and post-test knowledge. The level of knowledge was based on the score in pre-test and post-test, as mentioned in Table 1. Mean and standard deviations were calculated. A paired‑sample t-test was used to compare pre-and post-test scores. One‑way analysis of variance (F‑test) and independent student t-test was used to determine the association between demographic variables and mean pre-and post-test scores. To analyze the association within the groups with demographic variables, a post hoc test was used. A Chi-square test was used to check the association between knowledge level and participants’ demographic variables. Individual actual gains (Gi) were tabulated to calculate percent average actual gain (Gi = post-test score ‑ pre-test score) and percent relative gain (percent relative gain = average Gi /pre-test score) for the class [15]. This was intended to minimize dependence on the different levels of participants’ understanding prior to training. All the statistical tests were evaluated at the p < 0.05 level of significance.

## Results

The questionnaires through Google forms were sent to all participants before and after training, wherein 968 and 888 responses were received, respectively. Out of all responses, 575 participants, who filled both the pre- and post-test forms and gave consent to use their data, were included in the final analysis. The demographic details are shown in Table 1.

**Table 1:** Demographic variables of the participants in frequency (N) and percentage (%). HCW: health care worker.

		Frequency (N)	Percentage (%)
Age (mean 27.14, SD 3.07)	<=25	189	32.9
	26-30	322	56.0
	31-35	56	9.7
	>35	8	1.4
Gender	Male	317	55.1
	Female	258	44.9
Designation	Nursing Officer	570	99.1
	Residents	2	.3
	Technician	3	.5
Years of experience working as HCW	< 1	90	15.7
	1-5	402	69.9
	>5-10	71	12.3
	>10	12	2.1
Previous training regarding standard precautions for infection control	Yes	352	61.2
	No	164	28.5
	May be	59	10.3
Knowledge of standard precaution prior to training	Yes	470	81.7
	No	105	18.3
Have you ever used a virtual platform for training purpose	Yes	231	40.2
	No	344	59.8

**Figure 1:**
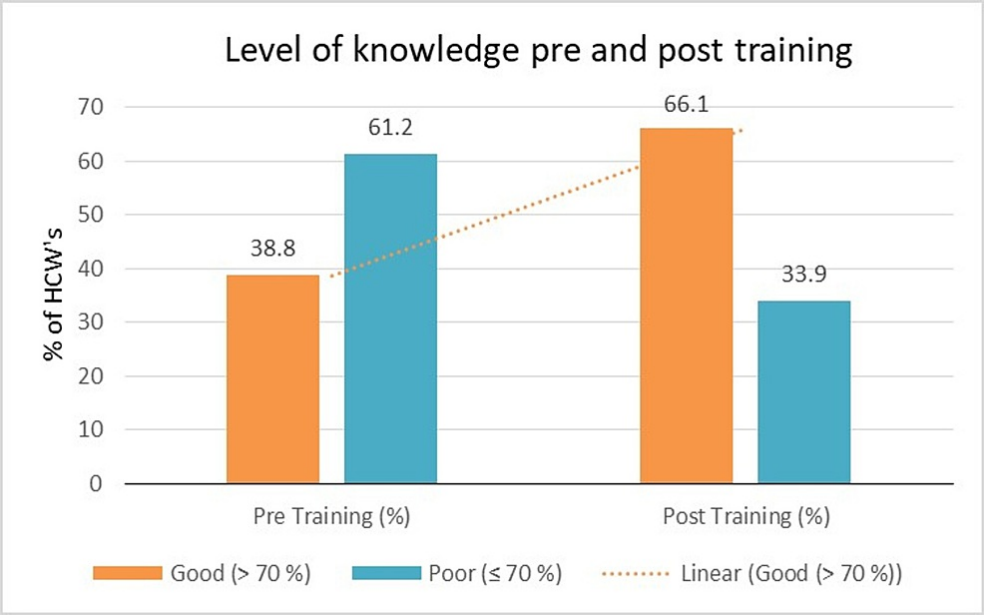
Pre- and post-training level of knowledge of participants. HCW: health care worker.

Although 61.2% of participants said they had received training regarding standard precautions, only 38.8% (223/576) had a good level of knowledge (Figure 1). The response rate for infection control-related questions with special emphasis on standard precautions asked before and after training is demonstrated in Table 2. There was a statistically significant gain in scores of all questions related to cleaning and disinfection; and Bio-medical waste. No significant change was observed in knowledge regarding the sequence of doffing (p-value: 0.941). The result of pairwise comparisons showed that there were statistically significant differences between pre-test and post-test scores (p-value < 0.001), as shown in Table 3.

**Table 2:** Pre-test and post-test response rate in various segments. PPE: personal protective equipment; BMW: bio-medical waste.

		Pre-training		Post-training		McNemar’s
	Question regarding	Correct answer Frequency (%)	Wrong answer Frequency (%)	Correct answer Frequency (%)	Wrong answer Frequency (%)	p-value
Do’s & Don’ts	Good ‘cough etiquette’	447 (77.7)	128 (22.3)	491 (85.4)	84 (14.6)	<0.001*
	Measures to prevent the spread of virus	540 (93.9)	35 (6.1)	550 (95.7)	25 (4.3)	0.100
Hand hygiene	Moments of hand hygiene	391 (68)	184 (32)	468 (81.4)	107 (18.6)	<0.001*
	Hand hygiene for unsoiled hands	254 (44.2)	321 (55.8)	347 (60.3)	228 (39.7)	<0.001*
	Sequence of hand hygiene	385 (67)	190 (33)	446 (77.6)	129 (22.4)	<0.001*
	Duration of hand rub	413 (71.8)	162 (28.2)	491 (85.4)	84 (14.6)	<0.001*
	Duration of hand wash	309 (53.7)	266 (46.3)	438 (76.2)	137 (23.8)	<0.001*
	Hand hygiene for visibly dirty hands	527 (91.7)	48 (8.3)	530 (92.2)	45 (7.8)	0.770
Personal protective equipment	Correct sequence of donning	327 (56.9)	248 ( 43.1)	430 (74.8)	145 (25.2)	<0.001*
	Correct sequence of doffing	428 (74.4)	147 (25.6)	496 (86.3)	79 (13.7)	0.941
	Removal of PPE	344 (59.8)	231 (40.2)	426 (74.1)	149 (25.9)	<0.001*
	Most critical step in PPE in donning/doffing	449 (78.1)	126 (21.9)	502 (87.3)	73 (12.7)	<0.001*
Cleaning and disinfection	Disinfectant for floor cleaning in COVID areas	271 (47.1)	304 (52.9)	362 (63)	213 (37)	<0.001*
	Responsibility for cleaning	336 (58.4)	239 (41.6)	430 (74.8)	145 (25.2)	<0.001*
	Disinfection of stethoscope	446 (77.6)	129 ( 22.4)	487 (84.7)	88 (15.3)	<0.001*
	Frequently touched non-metallic surfaces	202 (35.1)	371 (64.5)	241 (41.9)	334 (58.1)	0.007*
BMW	Discard of gloves	443 (77.0)	132 (23.0)	501 (87.1)	74 (12.9)	<0.001*
	BMW discard in quarantine	526 (91.5)	49 (8.5)	545 (94.8)	30 (5.2)	0.009*
	Body fluids/sputum disinfection	309 (53.7)	266 (46.3)	384 (66.8)	191 (33.2)	<0.001*

**Table 3:** Comparison of mean pre-test and post-test knowledge scores for different segments among HCWs. *Wilcoxon signed ranks rest applied. PPE: personal protective equipment; BMW: bio-medical waste; HCW: health care worker.

		Pre-test score	post-test score	Paired sample t-test
Segments	Maximum score	Mean ±SD	Mean ±SD	p-value (two-tailed)
Do and don't	2	1.72 ± 0.49	1.81 ± 0.41	< 0.001*
Hand hygiene	6	3.96 ± 1.41	4.73 ± 1.38	< 0.001*
PPE	4	2.69 ± 1.12	3.22 ± 0.99	< 0.001*
Cleaning and disinfection	4	2.18 ± 1.11	2.64 ± 1.14	< 0.001*
BMW	3	2.22 ± 0.73	2.49 ± 0.69	< 0.001*
Total	19	12.78 ± 3.13	14.89 ± 3.32	< 0.001*

**Figure 2:**
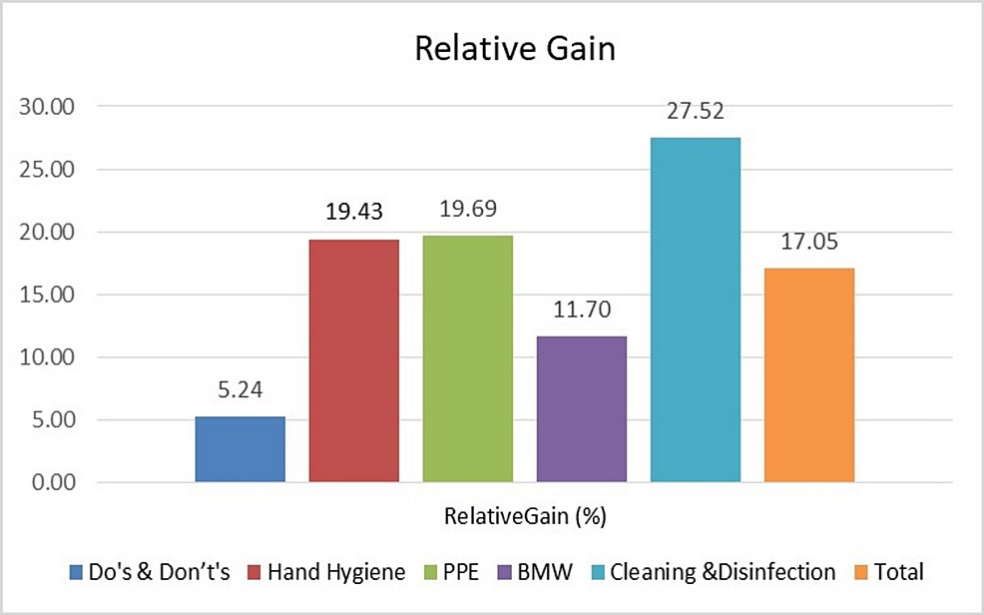
Relative gain (%) in knowledge scores in various segments. PPE: personal protective equipment; BMW: bio-medical waste.

There was a relative gain in knowledge scores in all segments with the maximum gain in knowledge (27.52%) in cleaning and disinfection segments. There was more than 19% gain in knowledge scores of hand hygiene and PPE domain (Figure 2).

**Figure 3:**
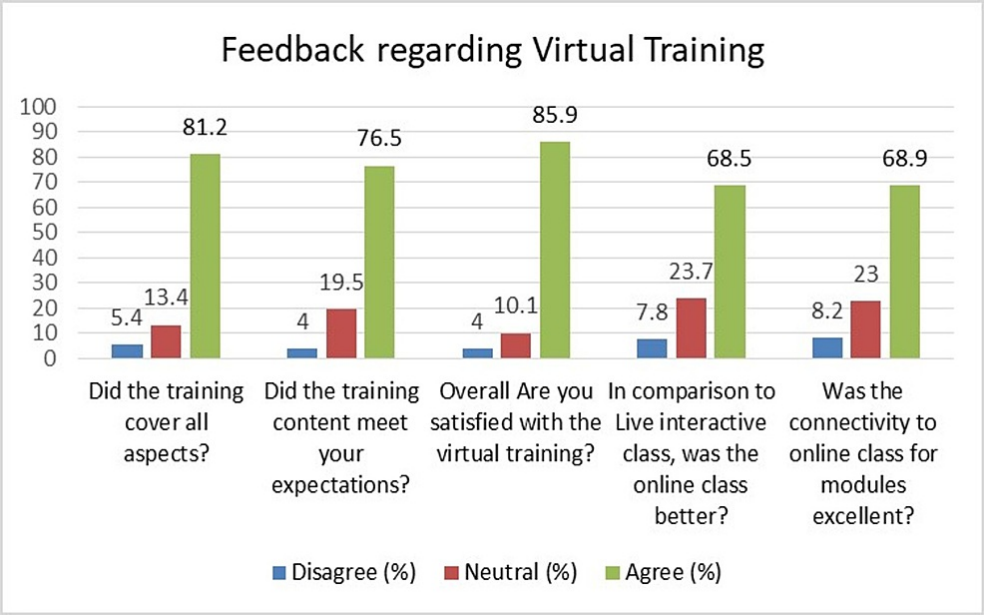
Distribution of feedback questions with each Likert scale of disagree, neutral and agree in number and percentages among HCWs. HCW: health care worker.

All the participants gave positive feedback regarding this new virtual training platform. More than 80% of participants were satisfied with the training (85.9%) and said that training covered all aspects of infection control-related topics relevant to protection from COVID-19 (81.2%). The distribution of feedback responses of participants given on the Likert scale design, depicted in Figure 3.

Problems faced during virtual training: Participants took time to familiarise themselves to get connected for the training sessions and during interaction like using chatbox to type questions to the trainer. Some participants had problems with connectivity due to poor bandwidth leading to frequent disconnections and poor transmission. Few participants kept two devices connected while some did not mute themselves leading to disturbance due to background noises.

## Discussion

Unlike the experience with previous epidemics (including Ebola), the COVID-19 pandemic has been characterized by the unprecedented global shutdown with varied restrictions being enforced by countries worldwide, adjusting and readjusting their response in accordance with the course of the pandemic. In the context of COVID-19, the infection control team played a pivotal role in the maintenance of essential healthcare services, by containing and preventing COVID-19 transmission within healthcare facilities to keep patients and HCW’s safe. All the HCW’s need to be well versed with the IPC practices, especially concerning COVID-19. The institute’s Hospital Infection Control Team (HICT) promptly planned and implemented multiple video-assisted live training sessions for the same [6]. Though live sessions were found to be effective, yet shift to an online platform of teaching was desired for adequate implementation of social distancing and to avoid gathering to prevent further transmission of the virus amongst HCW’s and yet not compromising on providing timely rapid training. Although direct teacher-student interactions are considered the foremost form of formal education yet as a result of the aftermath of the COVID-19 crisis, both students and teachers have been compelled to accept online education on different platforms. A few of the commonly used online platforms are Coursera, Zoom, Google Classroom, Blackboard Learn, Udemy, G Suite, and many more [16,17].

This study also highlights that lack of knowledge regarding PPE and donning and doffing which is one of the must-knows for all HCWs to combat COVID-19. Several published studies have focused on the use of PPE and hand hygiene compliance among HCWs. Despite the need for stringent IPC practices along with adequate use of PPE, researchers have observed that PPE compliance is passable and practiced procedures are varied among HCWs reflecting upon the requirements of IPC training for HCWs [7,18,19]. In a study by John et al., 9 % of HCWs reported that they had never received any formal training regarding PPE [20]. In our study, only 61.2 % of participants were sure of receiving prior training regarding infection control practices. Zellmer et al. observed that less than half (43 %) of HCWs removed PPE in the correct order [19]. These were similar to our study findings as very few HCWs in our study were familiar with the guidelines for donning/ doffing, and other IPC practices followed at our institute. Despite several tools and resources available at hand, compliance with PPE has been often found lacking among health care personnel, with many HCWs being unable to don and doff adequately, thus further increasing the risk of occupational exposures and illness [21]. Several studies in the past have reflected upon the need for infection prevention and control training [7,18,22,23]. This gave an insight into the significance of reinforcement training regarding IPC practices, including PPE training. Healthcare facilities, with the help of their HICT, must ensure the training of all HCWs regarding IPC practices for their as well as patients' better health.

Since long, several medical universities globally, including the Medical Council of India, have been encouraging colleges to use digital platforms and use technology to replace various disciplines. Even a few Indian medical institutes like St. John’s Hospital, Bangalore, and Christian Medical College, Vellore, use a software named TUSK platform to strengthen their distance learning programs for undergraduates and medical interns working in rural areas [24,25]. Yet online virtual platforms were not preferred over traditional live classes until the current COVID-19 pandemic. The pandemic has indeed taught all of us a lot and has brought the best out of us. Across the globe, the main mode of communication was chosen to be the internet or mobile phones, etc., to follow social distancing. Everyone, including teachers or students, has become skilled in using online educational platforms such as ZOOM, Cisco WebEx, Google Meet, etc., as a sign of positive transfer of learning. Also, some useful educational apps such as Office 365, Google classroom, and a much more user-friendly video conferencing app can be downloaded free of cost and easy to use [16].

In our study, we chose to reach our HCWs working in COVID areas through virtual platforms to brush up on their knowledge pertaining to IPC practices without any hitches. Video-based learning was attempted to reduce the number of trainers as facilitates This was the need of the hour, as with the incessant increase in COVID-19 cases, we had to assure the safety of our HCWs, and adequate training of all was of paramount importance. But, in the era of new normal with strict social distancing, virtual training platforms were the only options at our hands. We chose to use Zoom and Google meet platforms for the same. In our study, 66.1 % HCWs scored >70 % in the post-test compared to 38.8 % of participants scoring more than 70 %. With the help of our study results, we were able to find gaps in our training and specific training targets for future training. 

In-person face-to-face hand on training essentially requires trainer and trainee to be present at the same site [26]. COVID-19 pandemic posed a medical emergency like situation challenging the health care fraternity that required judicious use of HCWs to ensure a good number of workforce at a time for patient care, Due to its highly infectious nature physical distancing and discouragement of gathering was advocated, and hence the best possible option was delivering online training. However, even online training also requires a good pool of trainers. Therefore, video-based teaching was implemented by our Institute in which one resource person delivered the training using videos and interaction through online mode thereby ensuring a limited number of resource person and yet not compromising on content. The results of our study are quite encouraging and reflected the effectiveness of video-based training delivered through a virtual platform. Flipped classrooms have been applied by educationists in which resource material is given to learners and later face to face interaction happens in person to clear doubts and build some new information and facilitate the application of knowledge [27,28]. However, in our study, we did not provide any resource material to participants ahead of training. Video-based learning has been found to be effective in the acquisition of knowledge, skill, and attitude among HCWs [29].

This result of this study is quite encouraging as it reflects the efficiency of video-based learning through virtual mode. Within a short span of two months with minimal resource person training could be delivered to 968 participants in restrictive circumstances posed by the COVID-19 pandemic. The maximum effect in minimum time is the talisman to counter this pandemic, and online interactive training sessions may be considered an apt way to improve or reinforce knowledge about PPE as well as other infection control practices. In situations and circumstances that limit the availability of manpower resources to provide training to HCWs, the option of video-based education can be an effective means for imparting knowledge, skill, and attitude. Another advantage of video-based education, as also suggested by Duys et al., is that it can be circulated to be played and watched several times or the same video can be repeatedly be used to provide in-person or virtual training [26].

There were some limitations to this platform, such as some participants struggled with novel technology, participants with poor bandwidth connections, and background noises. Intermittent training through online mode can help trainees to get acquainted with virtual mode. Despite a few limitations, online platforms for infection control-related training, especially those pertaining to PPE and hand hygiene, have the edge over traditional physical classes during the current pandemic and are indeed of crucial help.

## Conclusions

Video-assisted teaching-learning through virtual platforms effectively trained health personnel on infection prevention and control practices during the COVID-19 pandemic. Video-assisted training can successfully be handled by a single resource person to impart the knowledge and skill to the trainee. Virtual teaching and learning is a feasible and efficient method to deliver training to HCWs on infection control practices and this methodology may be adopted in the future for several other training in manpower crunch situations and similar restrictive circumstances as posed by the COVID-19 pandemic. Furthermore, multi-centric studies, with participants from different institutes or hospitals may be planned in the future to assess the outcome among participants with different background knowledge. 

## Appendices

**Table 4:** Youtube link of video modules used for training.

S. No.	Video content	Link
1.	General instructions for healthcare workers	https://www.youtube.com/watch?v=uhmM4rdBcI0
2.	Do’s & Don’t’s	https://www.youtube.com/watch?v=xb_YP5P05es&feature=youtu.be
3.	Donning & doffing	https://www.youtube.com/watch?v=DLuLzQ9JDoQ&feature=youtu.be
4.	Cleaning & disinfection	https://www.youtube.com/watch?v=isAOyzGk8ak
